# Factors and common conditions associated with adolescent dietary supplement use: an analysis of the National Health and Nutrition Examination Survey (NHANES)

**DOI:** 10.1186/1472-6882-8-9

**Published:** 2008-03-31

**Authors:** Paula Gardiner, Catherine Buettner, Roger B Davis, Russell S Phillips, Kathi J Kemper

**Affiliations:** 1 Boston University Medical School, Department of Family Medicine, Boston, MA, USA; 2 Division of General Medicine and Primary Care, Beth Israel Deaconess Medical Center, Boston, MA, USA; 3 Department of Pediatrics, Public Health Sciences and Family and Community Medicine, Wake Forest University School of Medicine, Winston-Salem, North Carolina, USA; 4 Department of Family Medicine, Boston University Medical Center, 1 Boston Medical Center Place, Dowling 5 South Boston, MA 02118, USA

## Abstract

**Background:**

Little is known about the prevalence of dietary supplement (DS) use in American adolescents. We conducted this study to analyze the prevalence of DS use and factors associated with this use in a national population-based sample.

**Methods:**

We used data from the 1999 – 2002 National Health and Nutrition Examination Surveys (NHANES) for adolescents age 11 to 19. Using weighted logistic regression, we identified demographic and clinical factors associated with the use of any DS, vitamins or minerals, herbs and other DS.

**Results:**

Among the 5,306 responses representing approximately 36 million Americans 11–19 years old, 27% reported use of one or more DS in the prior month. The most commonly used DS were: multivitamins (16%) and vitamin C (6%). In the multivariable analysis, African American [adjusted odds ratio 0.40 (0.31–0.50) 95% CI] and Mexican American [0.55 (0.44–0.69)] adolescents were less likely to use DS compared with non-Hispanic whites. DS use was more common in those who used prescription medications [1.37 (1.10–1.72)] and among those who had a diagnosis of chronic headaches [1.25 (1.04–1.50)]. DS use was less common among those reporting fair or poor health status [0.59 (0.40–0.88)].

**Conclusion:**

Twenty seven percent of American adolescents use DS. DS use is higher among teens that use prescription medications; physicians and pharmacists should be aware of this, ask patients, and check for potential interactions.

## Background

The 1994 Dietary Supplement Health and Education Act (DSHEA) defined dietary supplements (DS) as vitamin, mineral, herb/botanical, amino acid, enzyme, protein, probiotic, glandular, or hormone-like substances [1]. Dietary supplements are used by more than 50 percent of American adults [2]. Use in adults is most common in those with higher income and education, women, and those with chronic health conditions. Clinical, regional, and internet-based surveys of adolescents have shown higher DS use among athletes and teens with chronic illnesses, but these associations need to be confirmed in population-based national surveys [3-9]. In small clinical surveys, pediatric chronic conditions for which herbal therapies and dietary supplements were reported include: attention deficit hyperactivity disorder (ADHD), asthma, atopic dermatitis, allergic rhinitis, cancer, inflammatory bowel disease, headache, and cystic fibrosis [3,10-18].

The 1988–94 National Health and Nutrition Survey (NHANES) found that approximately one fourth of adolescents used DS [19]. The 1999–2000 NHANES reported similar rates [20,21]. However, these analyses did not explore self-reported clinical conditions associated with teens' use of DS. [20,21]

We conducted this study to describe recent rates of DS use among US adolescents and factors associated with that use. Specifically, we wished to describe the prevalence of DS use among US adolescents age 11 to 19 and to analyze socio-demographic and clinical factors associated with DS use. Based on previous research, we hypothesized that DS use would be higher among: girls, older adolescents, non-Hispanic whites, those who diagnosed with common outpatient conditions, and those who reported using prescription medications.

## Methods

We used data from the 1999–2000 and 2000–2001 National Health and Nutrition Examination Surveys (NHANES) [22]. NHANES is a series of nationally representative surveys of the civilian, non-institutionalized population in the United States administered by the National Center for Health Statistics. The NHANES data are collected through household interviews and physical examinations at mobile examination centers. The sampling technique follows a complex, stratified, multi-stage probability design that over-samples adolescents, African Americans, Mexican Americans, and persons of low-income. NHANES has been conducted as a two year data collection with data released for public use in two-year increments. In 1999–2000 the household interview response rate was 82 percent, and in 2001–2002 the household interview response rate was 84 percent. Adolescents younger than 16 years old had a proxy (family member or guardian) answer for them unless they were an independent minor. Those 16 years and older answered the questions for themselves or had a proxy.

DS use was assessed from responses to questions asked during the household interview: "Have you used or taken any vitamins, minerals, or other dietary supplements in the past month?" Those who said "yes" were asked to provide dose, frequency, and duration of use and were asked to show the supplement containers to the interviewer so that supplement details could be recorded. If the container was not available (about 25 percent of the time), participants were asked for the exact name of the product, or to provide information in as much detail as possible. Additionally, respondents were shown a card during the interview with examples of dietary supplements including herbs, vitamins, minerals, fiber supplements, amino acids, fish oils, sport supplements, memory and energy promoting products. The survey did not ask about medicinal herbal teas, medicinal foods (nutraceuticals), or folk remedies.

Age, sex, race/ethnicity, total family income, physical activity, and health status were generally obtained from the head of household. We dichotomized the age variable into two age groups 11–15 years old versus 16–19 years old. Race/ethnicity was classified as non-Hispanic black, non-Hispanic white, Mexican American, and all others. Health status was assessed with the question "would you say your health in general is... excellent, very good, good, fair, or poor?" Respondents were asked if they used any prescription medications in past month.

Finally, to address the question as to whether adolescents with common out-patient conditions use more dietary supplements, we extracted data on chronic non-life threatening conditions frequently seen in out-patient practices asked about during the NHANES interview. Questions were asked about asthma, being overweight, and ADHD with the phrasing, "Has the doctor or other health professional ever told you that (you/he/she has) X ?". The conditions "hay fever in past year" and "frequent severe headaches in past year" were based on self report, not physician diagnosis. Many potentially clinically relevant conditions such as acne, depression, cystic fibrosis, and inflammatory bowel disease were not included in the survey.

### Statistical Analysis

We analyzed the data using SAS software (version 9.1; SAS Institute, Cary, NC) and STATA software (version 8; College Station, TX) due complex multistage design we used survey specific commands in the STATA statistical package which allowed incorporation of sampling weight strata, and primary sampling units to analyze the data. Sampling weights included adjustment for non-response, unequal probability of selection used in the sampling design. Because all data used in this study were from the interview portions of the surveys, we used the four-year interviewed sample weight for the combined analysis of NHANES 1999–2000 and NHANES 2001–2002 data [22].

We grouped the DS by category into vitamins/minerals or non-vitamins/minerals. Vitamins/minerals included: 1) any multivitamins/minerals (multivitamin/minerals or prenatal multivitamin); 2) any single vitamins or minerals. For non-vitamin/mineral DS use we included the following categories: 1) herbal products (e.g. echinacea, garlic); 2) non-herbal DS (e.g. probiotics, fish oil); and 3) appearance or performance enhancing supplements (e.g. protein powders or creatine). Some non-vitamin/mineral DS included vitamins as minor ingredients. In our efforts to capture the intended DS use by subjects, we used product labeling information and advertising materials to determine the category assignments.

We first assessed the associations between DS use and specific socio-demographic and clinical factors using the chi-square test. Next, we performed multivariable logistic regression to assess factors associated independently with use of any DS, any vitamin/mineral DS, and non-vitamin/mineral DS, while controlling for demographic, clinical factors, and specific outpatient conditions (asthma, ADHD, hayfever, headache, overweight). We selected variables for testing in our logistic model based on the results of previous studies and entered these variables into the final model simultaneously [8,20,23]. The variable income was removed from the model due to co-linearity with the variable race/ethnicity (Standard error for race/ethnicity increased by more than 25% when we added income to the model). Additionally, our original hypothesis questioned the association with race/ethnicity and dietary supplement use. Therefore, we keep race/ethnicity in the final model. However, since previous findings showed an association between income and DS use, we report descriptively the impact of income on DS use in the bivariate data (table 1) [24,25]. Results of multivariable models are presented as adjusted odds ratios with 95% confidence intervals. Due to multiple comparisons, statistical significance was set at a p value of 0.004 (Bonferroni correction).

**Table 1:** Characteristics of the 1999–2002 NHANES sample of US adolescents

*Demographic Factors*	N = 5306	Weighted Percent
Age		
11–15 years old	2919	56
16–19 years old	2387	44
Gender		
Female	2672	49
Male	2634	51
Race/Ethnicity		
Non-Hispanic White	1375	61
Non-Hispanic Black	1575	15
Mexican American	1956	11
All others	400	14
Family income		
< $15,000	1212	20
$15,000 – $34,999	1510	25
$35,000 – $64,999	1096	25
>$65,000	939	28
Refused or don't know	187	3
*Clinical Factors*		
Health status		
Excellent/very good	3320	71
Good	1515	23
Poor/fair	468	6
Prescription medication user		
No	4203	73
Yes	1101	27
Individual conditions		
Asthma	815	16
ADHD	356	9
Hayfever	583	13
Headache	1290	23
Obesity	650	10

## Results

There were 5306 participants from 11 to 19 years old, representing approximately 36 million US adolescents. Slightly more than half of respondents were in the 11 to 15 years old group and 49% were girls (Table 2). Most adolescents reported very good to excellent health (71%). Half of respondents reported one or more outpatient conditions; the most common condition was chronic headache (23%), followed by asthma (16%), hayfever (13%), obesity (10%) and ADHD (9%). More than one quarter (27%) of respondents had used prescription medications in the previous month (Table 2).

**Table 2:** Frequency of Dietary Supplements used by US adolescents (Weighted percents)

Type of Dietary Supplement	N	% users all ages 11 to 19 years	% users age11–15	% users age16–19	P value between ages difference
Any dietary supplements	1128	27	26	28	0.27
Any vitamin/mineral	961	23	23	23	0.85
Any Multivitamin	717	16	17	15	0.12
Non-prenatal	642	15	17	13	0.003
Prenatal	76	0.9	0.2	2	0.003
Single Vitamins/Minerals					
Vitamin C	222	6	6	7	0.43
Vitamin E	38	1	0.9	1	0.65
Vitamin B	35	1	0.8	1	0.50
Iron	28	0.5	0.1	0.9	0.004
Calcium	79	2	2	3	0.08

Any non-vitamin/mineral supplements*	185	4	4	5	0.06

Weight, sport, and enhancement supplements**	85	2	1	3	0.005
Herbal supplements***	80	2	2	3	0.39
Other non-herbal supplements****	36	1	1	1	0.25

*All non-vitamin/non-mineral supplements include: weight loss, sport, and other enhancement supplements, herbal and other DS.

**Weight loss, sport, and other enhancement supplements include: creatine, protein powders, stimulants, sport supplements, weight loss supplement, memory enhancers, or energy enhancing products.

***Herbal DS include: ginseng, gingko, garlic, echinacea, and other herbs.

**** Other non-herbal DS examples include: fish oil, probiotics, melatonin, glucosamine, and other non herbal non vitamin dietary supplements.

***** Chi Square test of independence

Overall, 27% of youth reported using one or more DS in the month prior to the survey. Non-vitamin/mineral DS were used by fewer than 5% of youth (Table 1). Older teens used more prenatal vitamins (p = 0.003) and iron (p = 0.004) than younger teens.

Frequency of using DS was increased in those with higher income. Non-Hispanic white youth used DS more commonly than minority youth (Table 3). DS use was highest among those reporting very good or excellent health and lowest among those with poor or fair health. Conversely, DS was higher among those who reported using prescription medications than those who did not. OverallDS use was slightly lower in youth diagnosed with asthma or overweight and higher among youth with ADHD, hay fever, or headache.

**Table 3:** Percentage of US adolescents using any DS, any vitamin/minerals, and other non-vitamin/mineral supplements by demographic and health factors

Characteristic	% using any DS N = 1128	% using any vitamin/minerals N = 961	% using non-vitamin/minerals N = 185
*Demographic Factors*			
Age			
11 to 15 years old	26	23	4
16 to 19 years old	28	23	5
Gender			
Female	28	24	4
Male	26	22	5
Race/Ethnicity			
Non-Hispanic White	32	27	6
Non-Hispanic Black	15	13	1
Mexican American	19	15	2
All others	22	21	3
Family income			
< $ 15,000	21	20	1
$15,000 – $34,999	20	16	4
$35,000 – $64,999	28	22	5
>$65,000	35	29	6
Refused/don't know	32	27	4
*Clinical Factors*			
Reported health status			
Excellent/very good	28	24	5
Good	24	21	4
Poor/fair	17	15	2
Prescription medication user			
No	24	20	4
Yes	33	29	5
Individual conditions			
Asthma	25	22	2
ADD	31	27	6
Hayfever	32	29	6
Headache	30	26	4
Obesity	25	19	5

In the a djusted multivariable model, certain demographic factors remained associated with DS use while others did not (Table 4). For example, non-Hispanic Black (0.40 [0.31–0.50] p = 0.000) and Mexican American (0.55 [0.44–0.69] p = 0.000) youth were less likely than non-Hispanic white to use DS. Older teens were more likely than younger teens to report using non-vitamin/mineral DS (1.71 [1.11–2.63] p = 0.016), but not more likely to use vitamin/mineral DS. On the other hand, after adjustments, gender was no longer associated with overall DS use or with any of the subcategories of DS use.

**Table 4:** Adjusted odds ratios and 95% confidence intervals for use of any DS, any vitamin/minerals, and non-vitamins/mineral use (N = 5250) *

Characteristic	Any DS	Any vitamin/minerals	Any non-vitamin/minerals
*Demographic Factors*			
Age			
11 – 15 years old	1.0	1.0	1.0
16 – 19 years old	1.11 [.92–1.33]	1.02 [.84–1.24]	**1.71 [1.11–2.63]**
Gender			
Female	1.0	1.0	1.0
Male	0.86 [0.73–1.02]	0.85 [0.69–1.02]	1.35 [0.93–1.96]
Race/Ethnicity			
Non-Hispanic White	1.0	1.0	1.0
Non-Hispanic Black	**0.40 [0.31–0.50]**	**0.44 [0.33–0.56]**	0.**27 [0.14–0.51]**
Mexican American	**0.55 [0.44–0.69]**	**0.58 [0.44–0.73]**	**0.44 [0.27–0.72]**
All others	**0.61 [0.44–0.85]**	0.76 [0.54–1.04]	0.47 [0.18–1.24]
*Clinical Factors*			
Health Status			
Excellent/very good	1.0	1.0	1.0
Good	0.89 [0.71–1.11]	0.89 [0.70–1.09]	0.83 [0.49–1.41]
Poor/fair	**0.59 [0.40–0.88]**	**0.61 [0.37–0.92]**	0.66 [0.23–1.89]
Prescription medication user			
No	1.0	1.0	1.0
Yes	**1.37 [1.10–1.72]**	**1.48 [1.16–1.90]**	1.07 [0.61–1.86]
Asthma			
No	1.0	1.0	1.0
Yes	0.84 [0.65–1.10]	0.90 [0.69–1.11]	0.49 [0.24–1.02]
Attention deficit disorder			
No	1.0	1.0	1.0
Yes	1.10 [0.82–1.49]	1.14 [0.82–1.53]	1.24 [0.65–2.39]
Hayfever			
No	1.0	1.0	1.0
Yes	1.23 [0.86–1.74]	1.26 [0.89–1.79]	1.46 [0.88–2.44]
Headache			
No	1.0	1.0	1.0
Yes	**1.25 [1.04–1.50]**	1.23 [0.99–1.46]	0.77 [0.42–1.40]
Obese			
No	1.0	1.0	1.0
Yes	0.86 [0.65–1.15]	0.80 [0.55–1.08]	**1.51 [1.07–2.13]**

Multivariable logistic regression: the variable income was removed from the model due to co-linearity with the variable race/ethnicity (Standard error greater then 25%) The original sample size was 5306. We excluded 56 subjects due to missing data. This is slightly more than 1% of the sample.

Similarly, in the adjusted model, some health factors remained associated with DS use while others did not. Compared with very good/excellent health, poor or fair health status was associated with a lower overall DS use (0.59 [0.40–0.88] p = 0.011). Conversely, adolescents reporting use of a prescription medication had higher overall rates of any DS (1.37 [1.10–1.72] p = 0.007) and vitamin/mineral supplements (1.48 [1.16–1.90] p = 0.003) use than those who did not use prescription medications.

After adjustment, the only specific condition that remained associated with a greater overall DS use was headache (1.25 [1.04–1.50] p = 0.018). Use of non-vitamin/mineral DS was more common among obese respondents (1.51 [1.07–2.13] p = 0.021).

## Discussion

This study describes the relationship between several varieties of DS use and clinical and socio-demographic factors in a nationally representative sample of American adolescents. Overall, 27% of adolescents reported using a DS in the last month. DS use was higher among non-Hispanic whites, those who reported better health status, and those who used prescription medications. However, we did not find the expected association between DS use and gender, nor after controlling for other factors, between DS use and any specific health condition other than headache. The findings that the most commonly used DS by teens were multivitamins (16%) and vitamin C (6%) is reassuring in terms of potential health risks. The higher use of non-vitamin/mineral supplements among obese teens raises questions about the types and safety of the specific DS used in this growing population.

The prevalence of DS use in our sample was consistent with that found in other large studies that used different sampling techniques [4,19,26]. The use of some non-vitamin/mineral supplements (e.g. sport and herbal performance enhancers) in this sample was lower than in studies of teen athletes [27-32]. It was also lower than rates reported in clinically based surveys [3,11,12,14,33]. Differences in rates might be due to different usage in different groups, differences in the time period of use, and by different survey methods such as self report using the Internet vs. parent or adolescent response in a face to face interview [4].

In the adjusted model, certain demographic factors were associated with supplement use, while others were not. We confirmed findings of another large survey in which older adolescents were more likely than younger teens to use non-vitamin/mineral DS such as herbals, sports and weight loss products [4]. Similarly, we confirmed earlier reports that non-Hispanic blacks and Mexican American adolescents use fewer vitamin/mineral DS than non-Hispanic white youth [4,19]. On the other hand, unlike earlier studies, after adjusting for other factors, female gender was not significantly associated with DS use [4,19]. Further studies are needed to understand how race, culture, income and health care disparities affect DS use and the impact of this use on health status.

As expected, certain clinical factors were also associated with DS use. For example, those reporting very good/excellent health status reported higher rates of DS use, confirming an earlier study [34]. The direction of this association (Does use of DS cause good health or are healthy teens more likely to use DS?) is a worthy question for future research.

As expected, we found a higher rate of DS use among teens who reported using prescription medications than those who did not, even after adjusting for potential covariates [23,24]. The etiology and clinical implications of the relationship between DS and prescription medication use requires additional exploration. Given reports about clinically serious interactions between prescription drugs and herbals/supplements, physicians and pharmacists need to be mindful of the higher use of DS among prescription users, ask prescription medication users about their use of DS, and monitor them for adverse effects or interactions.

The analysis of DS use by specific health conditions suggested mixed relationships. For example, obese youth were more likely to use non-vitamin/mineral DS than their peers. The higher use among obese teens is likely due to the inclusion of weight loss products in the non-vitamin/mineral DS variable; this finding deserves more specific scrutiny in future research for two reasons: a) rates of obesity are rising; b) weight loss products such as those containing ephedra can present risks of serious toxicity or drug-DS interaction. Also, adolescents with headaches were more likely to use DS, confirming earlier reports [35,36]. Future analyses are needed to look specifically at different types of DS with different disorders and the impact of DS use on disease-specific outcomes including use of professional health care, health status, quality of life, and ability to work or attend school.

Despite its large size, national scope, and extensive, detailed ascertainment of DS use, this analysis has several limitations. First, proxy information was used for respondents less than 16 years old. Proxies (e.g. parents) could have overestimated multivitamin use and under estimated use of products teens may have purchased on their own such as weight loss and performance-enhancing supplements. Second, questions about dietary supplements may have missed products considered home remedies, folk remedies, or parts of the diet, such as green tea or foods containing supplements. Furthermore, the limited data on specific health conditions reduced our ability to assess the complex relationships between diseases and DS use. Finally, since NHANES did not ask about reasons for supplement use or the impact of this use on health status, we do not know why teens used certain supplements or how it affected their health [37].

## Conclusion

DS use is most common among non-Hispanic white youth and those taking prescription medications; use of non-vitamin/mineral DS is most common among older teens and those who are overweight. Therefore, physicians and pharmacists should regularly ask patients about DS use, and check for potential interactions. To better understand use among culturally diverse groups and those with different clinical conditions, future studies should include a broader range of DS (such as those used in folk remedies, foods and medicinal teas) and ask about common health conditions. Additional studies are needed to determine the impact of DS use on health care use, health status and quality of life.

## Competing interests

The author(s) declare that they have no competing interests.

## Authors' contributions

PG crafted the study design, participated in the statistical analysis; interpreted the data; composed and revised the text. CB participated in designing the study, conducting the statistical analysis and reviewing the text. RD conducted the statistical analysis and reviewed the text. KK participated in designing the study, interpreting results, and composing and revising the text. RP participated in the study design, interpreting results, reviewing and revising the text. All authors read and approved of the final manuscript.

## Pre-publication history

The pre-publication history for this paper can be accessed here:


